# Erdheim‐Chester disease with long‐standing diabetes insipidus and generalized edema

**DOI:** 10.1002/ccr3.4898

**Published:** 2021-10-04

**Authors:** Faezeh Sadat Naji, Minoo Sadat Hajmiri, Zahra Mazari, Faeze Salahshour, Vahid Soleimani, Manouchehr Nakhjavani, Mahboobeh Hemmatabadi

**Affiliations:** ^1^ Department of Endocrinology Endocrinology and Metabolism Research Center (EMRC) Vali‐Asr Hospital Imam Khomeini Complex Hospital Tehran University of Medical Sciences Tehran Iran; ^2^ Cancer Institute Department of Pathology Imam Khomeini Complex Hospital Tehran University of Medical Sciences Tehran Iran; ^3^ Department of Radiology Imam Khomeini Complex Hospital Tehran University of Medical Sciences Tehran Iran

**Keywords:** diabetes insipidus, Erdheim‐Chester disease, non‐langerhans‐cell histiocytosis

## Abstract

Erdheim‐Chester disease (ECD) is a rare non‐Langerhans histiocytosis. ECD is detected more frequently due to increased awareness of healthcare providers and improved diagnostic tools. This report describes a 51‐year‐old woman with a history of weakness, bone pain, xanthelasma palpebrarum, and diabetes insipidus. ECD is a multisystemic condition with a poor prognosis. This disease should be considered in patients with diabetes insipidus, bone pain, and multiorgan involvements.

## Graphical abstract

Erdheim‐Chester disease is a rare and aggressive type of non‐Langerhans histiocytosis with an unknown etiology. This disease should be considered in patients with long‐standing diabetes insipidus, bone pain, and multiorgan involvements.

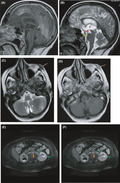

## 1 INTRODUCTION

Erdheim‐Chester disease (ECD) is a rare, non‐familial, and non‐Langerhans histiocytic disorder with an unknown etiology. It was named after describing two “lipoid granulomatosis” cases by William Chester and Jakob Erdheim in 1930.^1^ The real prevalence of ECD is unknown, but the number of reported cases has dramatically increased in recent years due to increased recognition of the disease.^2^ According to the multicenter 53‐patient case series by Arnaud L. et al^3^, it is more common between the ages of 40 and 70 years with a median of 57, and it is three times more prevalent among men.

Erdheim‐Chester disease is now considered a clonal hematopoietic disorder marked by recurrent MAPK pathway genetic alterations, with clinical manifestations caused by neoplastic histiocytic infiltrates and uncontrolled inflammation.^4^ A few attempts were made to determine the clonality of the non‐Langerhans cell histiocytes in ECD.^5^ ECD is diagnosed based on clinical manifestations and imaging features confirmed by histopathologic and immunohistochemical findings, such as positive CD68 and negative CD1a and S‐100.^4^, ^6^

Erdheim‐Chester disease initially affects the skeletal system, leading to an abnormal increase in bone density and fibrosis followed by severe pain. About 95% of the patients develop skeletal symptoms, in whom bone pain is the first symptom.^4^ The most common involved sites are the distal part of the femur and proximal parts of the tibia and fibula.^7^ Symmetric bilateral long bone lesions found on magnetic resonance imaging (MRI), and technetium‐99 m bone scintigraphy can be used to confirm the diagnosis.^8^ These lesions are also found in 18fluoro‐2‐deoxy‐d‐glucose (18FDG) positron emission tomography/computed tomography (PET)/CT scanning, but it is sometimes difficult to make a diagnosis based on plain radiographs.^9^ PET scan seems to be a proper choice for the assessment of disease activity because of the extra‐skeletal signs and symptoms of the disease. These extra‐skeletal involvements include diabetes insipidus, neurologic symptoms like cognitive impairment or seizure, pulmonary symptoms such as dyspnea or cough, cutaneous lesions and xanthelasma, pericardial effusion, and retroperitoneal involvement.^10^, ^11^

Currently, ample evidence supports the use of interferon‐α (IFN‐α) and Pegylated IFN‐α as the first‐line therapies for the disease. Other medications such as anakinra, an anti‐cytokine agent, are effective, especially in patients without CNS involvement.^2^

Although the 5‐year survival of ECD patients treated with IFN‐α is reported to be 68%, its prognosis is considered poor overall.^2^

This paper presents a patient with ECD who had a history of prolonged diabetes insipidus (DI), generalized edema, and xanthomas. The clinical presentations of this disease and its clinical course and treatment are further discussed.

## 2 CASE REPORT

**FIGURE 1:**
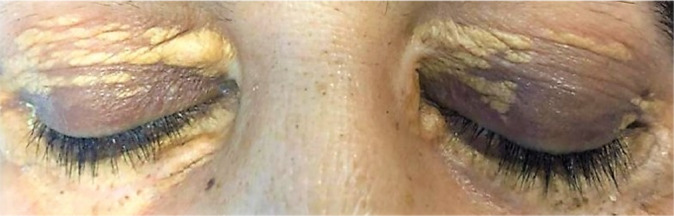
Photograph of the eyes revealing Xanthelasma palpebrarum

A 51‐year‐old woman was admitted to our endocrinology ward with a history of weakness, anemia, bone pain (since 2 years ago), xanthelasma palpebrarum (Figure 1), ulcerative skin lesions (since 5 years ago), and progressive generalized edema (since 1 month ago). She had DI since 12 years ago that was treated with desmopressin. She also received levothyroxine for hypothyroidism. The patient was aware and awake with normal vital signs. Thyroid examination was remarkable for a right lobe nodule, deep tendon reflexes of lower limbs were absent, and the left lower limb had a decreased muscular force (3/5).

Laboratory findings revealed anemia (Hb = 9.1 g/dl), elevated serum alkaline phosphatase (155 U/L), and lactate dehydrogenase (174 U/L), high 24‐h urine protein (594 mg), and low serum albumin (2.2 g/dl), and total protein (3.8 g/dl). Serum creatinine, electrolytes, and serum lipid profile were in the normal range except for serum triglycerides of 260 mg/dl. Moreover, anterior pituitary hormones were within the normal range except for a prolactin level of 1447 Miu/ml.

Since hypoalbuminemia and proteinuria were lower than the nephrotic range, upper and lower endoscopies were performed to rule out protein‐losing enteropathy, which were normal. Unfortunately, the patient did not agree to push enteroscopy.

A thyroid ultrasound showed a 3*3.2 mm non‐calcified nodule in the left lobe and a 48*50*73 mm heterogeneous solid mass with calcified foci in the right lobe. Pathological examination of the right lobe mass fine‐needle aspiration reported a benign follicular lesion and *BRAF*‐*V600E* mutation was negative in the Immunohistochemistry study.

**FIGURE 2:**
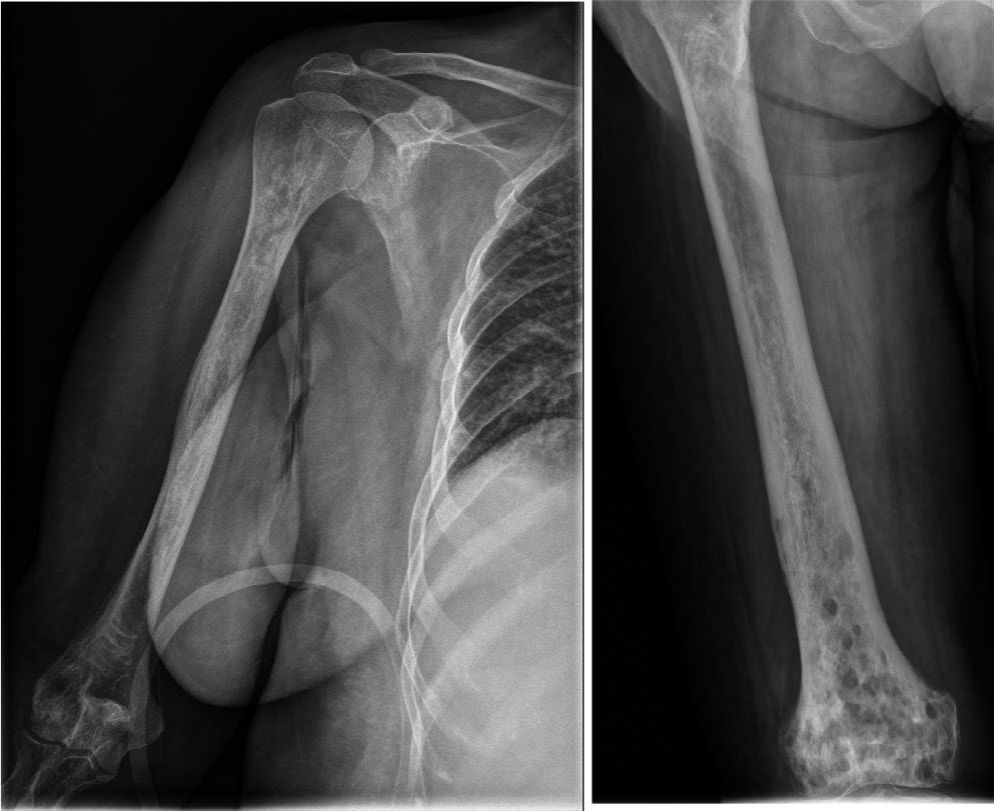
Humeral and femoral plain X‐ray revealing bilateral symmetric metaphyseal—diaphyseal sclerosis

Humeral and femoral plain X‐ray showed bilateral symmetric metaphyseal‐diaphyseal sclerosis. (Figure 2).

A chest CT scan revealed a mild pericardial effusion, bilateral pleural effusion, few mediastinal lymph nodes (SAD = 10 mm), a thyroid nodule (35 × 37 mm with calcification), and small sclerotic lesions in bilateral humerus meta‐diaphysis. In echocardiography, mild pericardial effusion and normal LV systolic function were reported.

An MRI examination of the brain showed an empty sella and flattening of the pituitary gland over the sellar floor. Abnormal enhancement of the pituitary stalk was obvious with a maximum thickness of about 6 mm. There was also severe abnormal thickening and enhancement of the tentorium cerebelli with a maximum thickness of about 17 mm.

**FIGURE 3:**
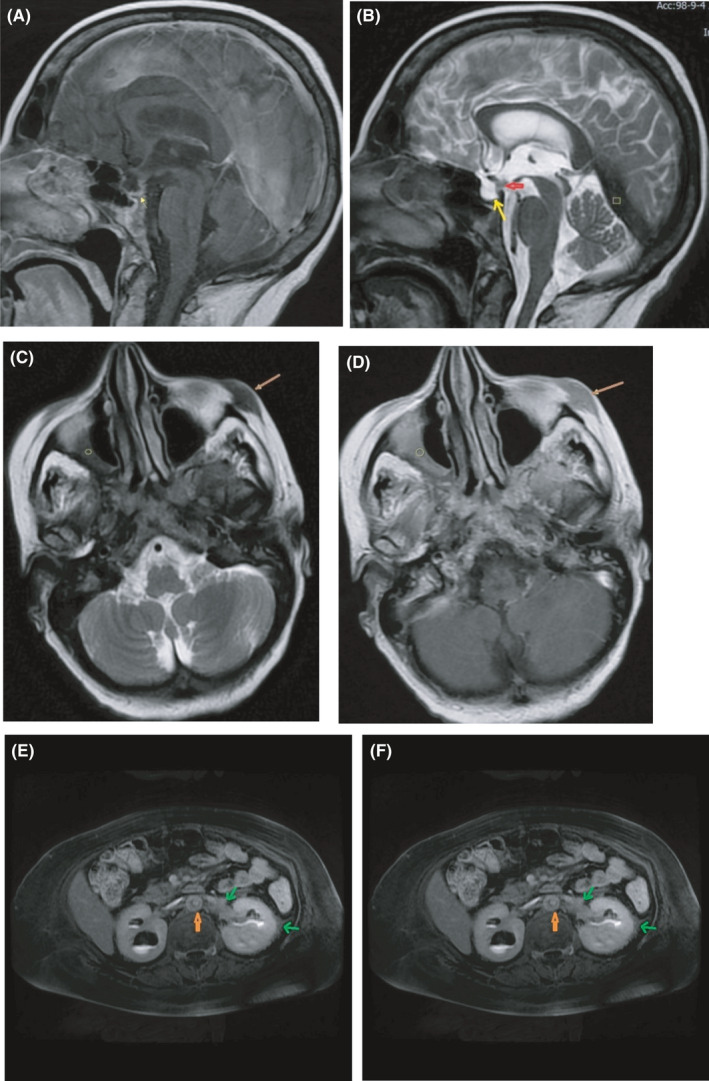
A and B, sagittal T2‐weighted and coronal contrast‐enhanced T1–weighted images of brain show low T2 signal, enhancing thickening of tentorium cerebelli and falx cerebri (rectangle) along with empty sella and flattening of compressed pituitary gland along sellar floor (thin yellow arrow) and faint enhancing thickening of pituitary stalk (thick red arrow).C and D, axial T2 and post‐contrast T1–weighted images of skull base show low T2 signal,enhancing soft tissue in right maxillary sinus (circle)and left cheek subcutaneous fat (brown arrow) E and F, axial T2 FIESTA and post‐contrast T1–weighted images of abdomen show low T2, enhancing soft tissue around abdominal aorta faintly infiltrate along renal vascular pedicle, renal sinuses, and perinephric spaces leading to bilateral hydronephrosis

Axial T2 and post‐contrast T1–weighted images of the skull base demonstrated a low T2 signal, enhancing soft tissue in the right maxillary sinus, and left cheek subcutaneous fat. In fact, axial T2 FIESTA and post‐contrast T1–weighted images of the abdomen showed low T2, enhancing soft tissue around the abdominal aorta faintly infiltrating along the renal vascular pedicle, renal sinuses, and perinephric spaces leading to bilateral hydronephrosis. (Figure 3).

**FIGURE 4:**
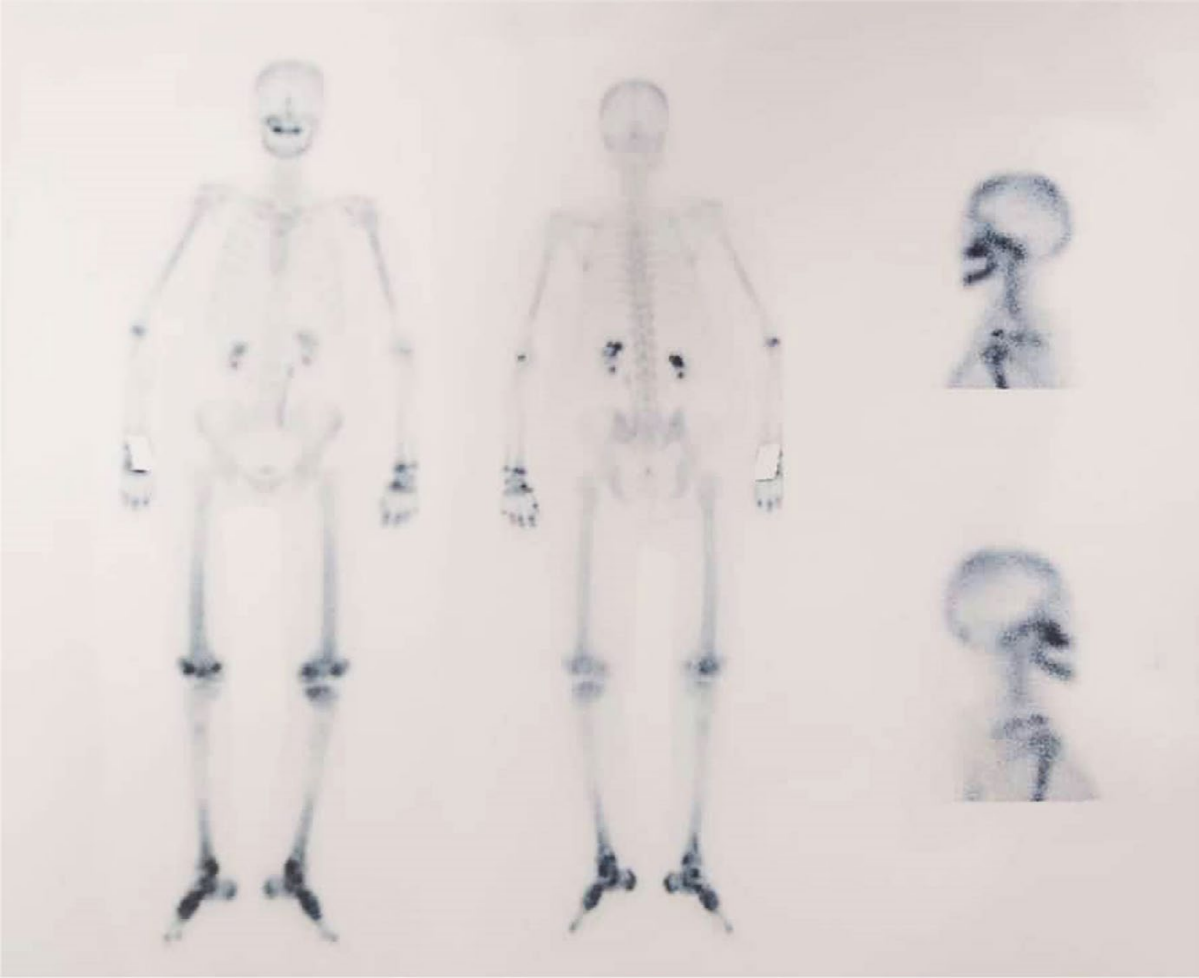
Whole body bone scan revealing symmetrical and diffused increased radiotracer uptake

A whole‐body bone scan revealed symmetrical increased radiotracer uptake in the mandible, pelvic bones, bilateral femora, bilateral tibiae, and metatarsal bones. There was also a focus of activity in the proximal right tibial diaphysis. Myocardium plus pericardial space shadow was thickened. A bilateral dilated pyelocaliceal system was also noted. (Figure 4).

A bone marrow biopsy revealed 40% cellularity as well as decreased trilineage population of hematopoietic elements that were replaced by histiocytic infiltrates exhibiting abundant foamy cytoplasm and bland‐looking nuclei. Scattered eosinophils were also noted.

**FIGURE 5:**
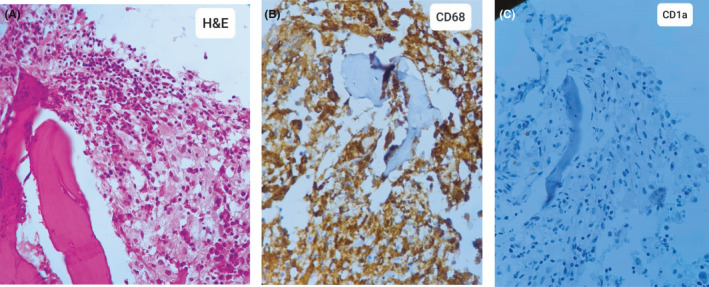
A, Hematoxylin‐eosin–stained Bone marrow biopsy revealing lipid‐laden histiocytes, Histiocytic infiltrate exhibiting abundant foamy cytoplasm, and bland‐looking nuclei B, IHC stain for CD68 revealing positivity of histiocytes and C, negativity for CD1a

Flow cytometric Immunophenotypic study showed cellular bone marrow with reactive changes and a marked shift to the left at myeloid series. Immunohistochemistry study results showed CD68 (+, diffuse), Fascin (+), MPO (highlights myeloid series), CD20 (occasional scattered positive), CD3 (occasional scattered positive), TDT (no excess of blast), CD34 (no excess of blast), CD1a (‐), S100 (occasional scattered positive), and Ki67 (+). (Figure 5).

Therefore, considering the consensus diagnostic criteria for ECD, the diagnosis was confirmed due to xanthelasma palpebrarum, bilateral and symmetric skeletal abnormalities in the diaphyseal and metaphyseal regions of the long bones, and bone marrow biopsy findings of foamy CD68(+)/CD1a(‐) histiocytes, often with admixed inflammation and fibrosis.

Vinblastine 1 mg/m^2^ weekly and prednisolone 50 mg daily were prescribed for the patient for 8 weeks. Following the stability of the patient's condition and improvement of the symptoms, she was discharged after 2 weeks of treatment but she was still ill. Unfortunately, she died in her town a few days after discharge due to acute pulmonary edema.

## 3 DISCUSSION

This study reports a case of Erdheim‐Chester disease. ECD is a rare non‐Langerhans histiocytic disorder with systemic manifestations ranging from asymptomatic disease to fulminant multiorgan damage. Our patient had diabetes insipidus for a long time as the only symptom of the disease, and hence, the diagnosis was delayed. However, unusual tissue presentations and the insidious onset of this rare disease often result in a diagnostic delay. On admission, complementary investigations were done due to the presence of xanthelasma and skeletal symptoms and the results led us to the diagnosis. According to the literature, the involvement of long bones is the only specific presentation of ECD, in which the long bones of lower extremities are more commonly involved compared to the long bones of upper extremities; however, up to 50% of the patients may have asymptomatic bone lesions.^12^ Neurological involvement is the most common presentation after the skeletal system, which can be in the form of diabetes insipidus, cerebellar ataxia, panhypopituitarism, and papilledema. Half of the ECD patients have extra‐skeletal manifestations. The most common extra‐skeletal manifestations include diabetes insipidus, hypothyroidism, and skin involvement which were found in our case.^13^ Other involved organs in order of prevalence include cardiovascular (pericardial pain, cardiac tamponade, cardiac failure, and myocardial infarction), renal (dysuria, abdominal pain, chronic kidney failure, and renovascular hypertension), skin (xanthelasma), ocular (exophthalmos, diplopia, and visual impairment), constitutional (fever, fatigue, and weight loss), pulmonary (cough and chest discomfort), and endocrine system (hyperprolactinemia, gonadotropin insufficiency, and IGF deficiency).^14^ Cardiovascular infiltration is reported in 40% of ECD cases. Periaortic fibrosis is the most frequent vascular involvement with sometimes a "coated aorta" aspect. Other arteries may also be infiltrated in this disease, including renal artery stenosis or life‐threatening ischemic manifestations like myocardial infarction or mesenteric angina. Some patients may present with pericarditis possibly leading to tamponade.^15^ Our patient had a mild pericardial effusion and bilateral hydronephrosis.

The available literature recommends corticosteroids, cytotoxic agents, and immunosuppressive drugs as promising treatments for ECD patients.^2^, ^16^ IFN‐α and Pegylated IFN‐α have been introduced recently with promising results, and response to this treatment is the only major predictor of survival particularly in patients with CNS involvement.^16^ Several treatment options such as targeted therapy against BRAF, IL1 antagonists, and inhibitors of MEK, TNFα or tyrosine kinases have been also used. According to the latest consensus recommendations, BRAF‐inhibitor therapy with vemurafenib or dabrafenib can be considered for patients with BRAF‐*V600E* ECD who have cardiac/neurologic disease or end‐organ dysfunction and BRAF‐inhibitors or conventional therapy can be used for patients with BRAF‐*V600E* ECD without end‐organ dysfunction. Empiric treatment with MEK‐inhibitor should be strongly considered for ECD patients without BRAF‐*V600E* and cardiac/neurologic disease or end‐organ dysfunction.^17^ Our patient was treated with vinblastine 1 mg/m^2^ weekly and prednisolone 50 mg daily due to availability and experience.

The prognosis of this histiocytosis is relatively poor. Common causes of death include cardiac failure and pulmonary fibrosis.^13^ Our patient was in relatively good condition after starting the treatment; edema reduced moderately and the patient was discharged from the hospital while she was still ill. Unfortunately, she died from acute pulmonary edema in another town within a few days. We could not find more details.

In conclusion, ECD is a very rare non‐Langerhans histiocytosis. The cause and pathogenesis of this disorder remain unclear, and further studies are warranted. It has pathognomonic radiographic features and pathological evaluation shows lipid‐storing CD68 (+), S100 (variable), CD1a (−) histiocytes. ECD is a multisystemic and heterogeneous clinicopathological condition with a poor prognosis. This disease should be considered in patients with diabetes insipidus, especially when there is bone pain and multiorgan involvements.

## CONFLICTS OF INTEREST

The author(s) declared no conflicts of interest with respect to the authorship and publication of this article.

## AUTHOR CONTRIBUTIONS

Mahboobeh Hemmatabadi and Manouchehr nakhjavani involved in study concept and design, and critical revision of the manuscript for important intellectual content. Faezeh Sadat Naji, Minoo Sadat Hajmiri, Zahra Mazari, Faeze Salahshour, and Vahid Soleimani involved in analysis and interpretation of data. Faezeh Sadat Naji and Mahboobeh Hemmatabadi involved in drafting of the manuscript.

## Data Availability

Data available on request due to privacy/ethical restrictions.
